# Psychological Burden and Communication Challenges Among Relatives of Older Patients With Dementia—A Cross-Sectional Study in an Acute Psychiatric Hospital

**DOI:** 10.1177/07334648251372691

**Published:** 2025-09-08

**Authors:** Giulia Zerbini, Emanuel Wiese, Laura Muehlich, Alkomiet Hasan, Miriam Kunz, Jan Haeckert, Philipp Reicherts

**Affiliations:** 1 Department of Medical Psychology and Sociology, Faculty of Medicine, Institute of Theoretical Medicine, 531257 University of Augsburg, Germany; 2 Department of Psychiatry, Psychotherapy and Psychosomatics, Faculty of Medicine, Bezirkskrankenhaus, 531257 University of Augsburg, Germany; 3 DZPG (German Center for Mental Health), Partner Site München, Augsburg, Germany

**Keywords:** informal caregivers, relatives of patients with dementia, burden, depression, communication, psychiatric hospital

## Abstract

Research on the relatives’ well-being during the critical time point when their family member is hospitalized in an acute psychiatric hospital is still lacking. Therefore, we investigated psychological well-being, care-related burden, and communication challenges of 67 relatives of older patients with dementia (RPwD) versus 60 relatives of older patients with a psychiatric disorder (RPP) at the time of hospitalization. RPwD reported significantly higher levels of depression and care-related burden compared to RPP (there was a similar non-significant trend for anxiety). RPwD reported more communication problems with the patients, while they appeared more satisfied regarding the communication with the clinical staff. Both groups reported similar levels of stress and resilience. The present study extends previous findings demonstrating that taking care of an older relative with dementia, but also with a psychiatric disorder, is a great burden. Professional support to maintain the well-being of relatives of older patients is recommended.

**What this paper adds**

• Relatives of patients with dementia are not only more psychologically burdened compared to age matched controls, as previously shown, but also compared to relatives of psychiatric patients, another group showing elevated levels of burden.
• How psychological burden of relatives changes when patients are hospitalized has often been overlooked. Here we show that the psychological burden of relatives of patients with dementia, but also with a psychiatric disorder, remains elevated during this critical time.

**Applications of study findings**

• The findings of this study underscore the importance of providing ongoing psychological support to the relatives of older patients, as their psychological burden persists even during periods when caregiving duties are paused, such as during the patient’s hospitalization.
• Depressive symptoms, care-related burden, and communication challenges with the patient seem to be the most critical aspects that should be addressed to improve the well-being of relatives of patients with dementia.

## Introduction

Life expectation is continuously rising and by that the ratio of individuals who are older than 65 years in the general population is increasing (Knecht et al., 2023). Along with this socio-demographic trend, the prevalence of patients living with neurodegenerative diseases is also increasing (Alzheimer-Europe, 2019; Matsuoka et al., 2019; Mukadam & Sampson, 2011; Wolters et al., 2020). According to the World Health Organization (WHO), 55 million people are diagnosed with dementia worldwide and this number will almost triple by 2050, making dementia an urgent health priority. The heterogeneous symptoms of dementia including cognitive decline and behavioral changes are not only very debilitating for the patients, but also one of the major causes of burden for their relatives (García-Martín et al., 2023). Relatives of patients with dementia (RPwD), especially if they are also the informal caregivers, represent a vulnerable population, showing psychological and physical health problems and increased levels of physiological stress markers (Vitaliano et al., 2003). Nearly 30% of informal caregivers responsible for persons with dementia experience depression, while around 50% perceive their caregiving duties as burdensome (Cheng, 2017; Collins & Kishita, 2020; Wulff et al., 2020). The level of burden is not only increased compared to matched controls, but also higher compared to relatives of patients with psychiatric disorders (Cham et al., 2022; Connors et al., 2020). This is probably because the care of dementia patients is very time-intensive and resource-demanding, given the characteristics of their disease (e.g., memory and language deficits, but also changes in emotional and behavioral responses when the disease is progressing) (Watson et al., 2012).

While there is a large body of literature on patient relatives’ burden in general (Cheng, 2017; Collins & Kishita, 2020; Etters et al., 2008), only few studies have investigated their well-being when the patients are hospitalized in an acute psychiatric hospital, despite the observation that older individuals with dementia are more likely to get hospitalized than older individuals without dementia and that hospital stays are usually longer for this population (Möllers et al., 2019; Shepherd et al., 2019). A European study showed that 26.15% of the patients admitted to psychiatric hospitals needed treatment because of symptoms related to a dementia disease (Takacs et al., 2015). Here, the main symptoms leading to a psychiatric hospitalization were aggressive behavior, delirium, aimless divagation and confusion (Takacs et al., 2015). Additionally in a German study, 81.8% of the geronto-psychiatric admissions were due to behavior endangering themselves or others (Wetterling et al., 2008). In addition to psychological burden, dementia-related symptoms can lead to an experienced violation of the RPwDs’ inner value and thus dignity, as a recent qualitative study from our group has shown (Wiese et al., 2025).

When patients with dementia (PwD) are admitted for psychiatric treatment, and thus are not anymore under the care of their relatives, the psychological burden can still be present, or become even worse (Epstein-Lubow et al., 2012). After years of acting as caregivers, the relatives’ burden might in fact reach a peak in conjunction with the psychiatric hospitalization or institutionalization, which usually reflects a significant worsening of the patients’ clinical picture (Epstein-Lubow et al., 2012; Gaugler et al., 2010). It is thus essential to support the relatives also during this critical phase, yet research about the psychological burden among RPwD around the time point of hospitalization is lacking.

The involvement of the relatives in the treatment decisions and the quality of the communication with the clinical staff appear important aspects for the relatives’ well-being. For instance, increased caregiver-satisfaction has been reported if the relatives are included in the decisions about treatment and in the care of the patients (Keuning-Plantinga et al., 2021). Ineffective communication between the relatives and the clinical staff can, on the contrary, result in additional burden (Keuning-Plantinga et al., 2021).

The aim of the present study was to investigate the well-being of RPwD during the critical time point when patients are admitted to a psychiatric hospital. We assessed psychological well-being, care-related burden of RPwD at the time when their relative was hospitalized in an acute psychiatric hospital and compared it with a group of relatives of older patients with a psychiatric diagnosis (RPP), who also had been recently hospitalized. Furthermore, communication challenges that relatives typically experience with the patients and with the clinical staff, respectively, were assessed and compared across the two groups.

## Methods

Data were collected between the 2^nd^ August 2022 and the 19^th^ October 2023. The study was approved by the ethics committee of the medical faculty at the Ludwig-Maximilians-University (LMU) Munich and the Medical Faculty of the University Augsburg (project number 22-0430) and was conducted in accordance with the Declaration of Helsinki.

### Participants

Participants were relatives of geronto-psychiatric patients (older than 65 years) who had been recently hospitalized in the geronto-psychiatric acute care unit of the Department of Psychiatry, Psychotherapy, and Psychosomatics of the University of Augsburg (Germany), which is a psychiatric hospital where the full spectrum of psychiatric diseases are treated. The hospital includes three specialized geriatric psychiatry wards, providing a total of 66 inpatient beds. In 2024, the average length of stay in these geronto-psychiatric wards was 30.3 days (±8.0 days), depending on the complexity of the diagnosed condition. Admissions occur either on a voluntary basis or, when necessary, involuntarily in accordance with legal provisions. Upon discharge, patients either return to their private residences or are (re-)transferred to long-term care facilities. Occasionally, due to the patients’ limited capacity to provide informed consent, caregiving relatives act as proxies. In Germany, psychiatric hospitals provide care across the full range of mental disorders classified within ICD-10 categories F1X to F9X.

**Figure 1.**
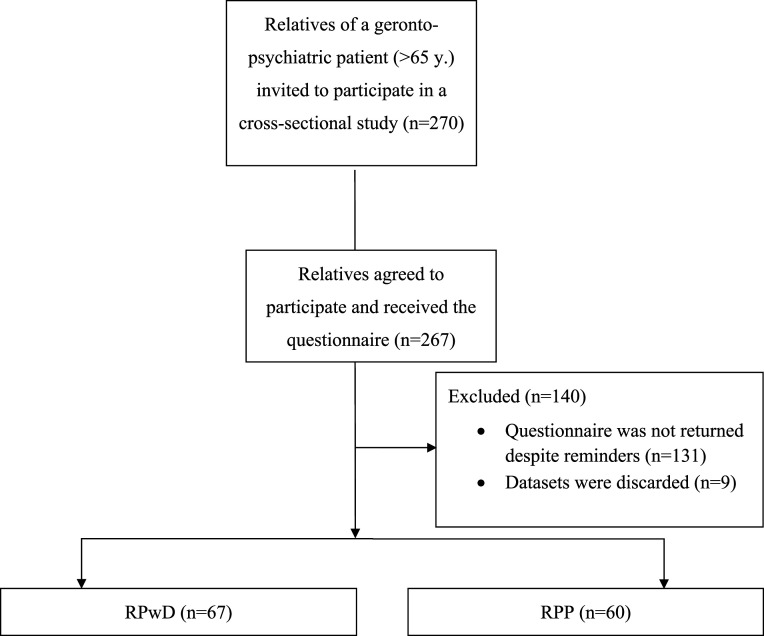
CONSORT Diagram Illustrating the Study Process. *Note:* RPwD = Relatives Patients With Dementia; RPP = Relatives Patients Psychiatry

Participants were recruited via telephone. In total, 270 participants were contacted, 267 agreed to participate and received the questionnaires via mail, 136 surveys were sent back. Based on their self-reports, participants were assigned to the groups “relatives of patients with dementia” (RPwD) or “relatives of psychiatric patients” (RPP). We did not collect data from an age-matched control group of participants that are not involved in the care of a relative, see also the limitation paragraph. Nine participants did not report the diagnosis of their relatives and thus these datasets were discarded from further analyses (final sample size *N* = 127; Figure 1). All participants gave their informed consent, and they did not receive any monetary compensation.

### Questionnaires and Scales

We asked participants to fill in a series of questions assessing sociodemographic characteristics, including details on their relationship to the patient and the amount of care work, for further details please see Table 1. In addition, participants were asked whether their relative was diagnosed with dementia by a medical doctor (yes or no), whether their relative was diagnosed with a psychiatric disorder (yes or no), and what the exact diagnosis was (open ended questions). Group allocation—RPwD versus RPP—was based on the information provided by the participants. Furthermore, we assessed the relative’s psychological well-being, their care-related burden, their resources, that is, resilience, how they experienced difficulties regarding the communication with their relatives and the communication and contact with the staff in the clinic.

**Table 1.** Sample Characteristics (N=X)

Measure/Indicator	RPwD	RPP
Number of participants	*n* = 67	*n* = 60
Age, mean (SD)	59.05 (11.16)	61.51 (12.02)
Sex: Female/male (not answered)*	49/18 (0)	24/35 (1)
Employed: Yes/no (not answered)	36/26 (4)	34/22 (3)
Marital status		
Single	4	8
Married	46	38
In a relationship	9	4
Divorced	5	7
Widowed	2	0
Separated	1	1
not answered	0	2
Relationship to the patient		
Partner	15	20
Child	43	32
Daughter-in law/Son-in-law	3	0
Else or (not answered)	6	7
Living together with patient: Yes/no (not answered)*	17/50 (0)	27/32 (1)
Other relatives involved in care yes/no (not answered)*	43/22 (2)	25/30 (5)
Patient sex: female/male*	34/33	46/14
Patient age: mean years (SD)*	80.79 (7.09)	78.18 (6.80)
Time per day spent with care: mean h (SD)	5.09 (5.85)	3.88 (6.24)
Care level (= the local measure of the level of care or assistance a person requires due to illness or disability, ranging from 0 [no or almost no care needed] to 4 [full need for care])*		
0	6	28
1	9	4
2	12	13
3	26	8
4	13	2
Not answered	1	5
Years since patient needs professional care: mean (SD)	2.56 (2.01)	3.57 (5.00)

*Note.* *group comparison revealed a significant difference (*p* < .05).

#### Mental Health—Patient Health Questionnaire (PHQ)

The PHQ is a widely used questionnaire to assess general mental health (Beard et al., 2016; Kroenke et al., 2001). Here, we used three subscales assessing depression (PHQ-D, nine items), stress (PHQ-S, nine items), and anxiety (PHQ-A, seven items). The PHQ-D subscale ranges between 0 and 27, the PHQ-A subscale ranges between 0 and 21, the PHQ-S subscale ranges between 0 and 18. Higher scores indicate more severe symptoms.

#### Zarit Burden Interview Assessing Caregiver Burden (ZBI)

The ZBI is a questionnaire designed to assess caregiver burden (Seng et al., 2010; Zarit et al., 1980). We used the revised version with 22 items with response options between 0 (never) and 4 (nearly always). The sum score ranges between 0 and 88 with higher scores indicating more burden.

#### Resilience–Connor-Davidson Resilience Scale (CD-RISC-10)

The CD-RISC-10 is a short version of the original CD-RISC scale to assess resilience (Campbell-Sills & Stein, 2007; Connor & Davidson, 2003; Sarubin et al., 2015), with response options between 0 (not true at all) and 5 (always true). The sum score ranges between 0 and 50 with higher scores indicating more resilience.

#### Communication Challenges with the Patient

To assess communication problems with the patients, we asked participants to fill in an adapted subscale of the *Berliner Inventar zur Angehörigenbealstung*—*Demenz (BIZA-D)* [Berlin relatives of patient with dementia burden inventory], Modul 2 Verhaltensstörungen des demenzerkrankten Angehörigen [Modul 2 Behavior Disorders of the relative suffering from Dementia] comprising five items (Schacke & Zank, 2009). Items describe common problems about the communication with the patient (e.g., the patient often repeats themselves), providing response options on a seven-point scale, ranging from 0 (not at all) to 6 (extremely). A mean score was calculated for the items that were answered by the relatives (all items had the responses option “not applicable”). Higher scores indicate more communication problems with the patient.

#### Evaluation of Communication Quality with the Clinic Staff

To assess how relatives experience the communication with the clinic staff, participants were asked 10 questions, slightly adapted in wording to reflect the acute psychiatric setting, which were taken from the modul *Kommunikation* [communication] of the *Angehörigenbefragung in der stationären Langzeitpflege* [Questionnaire for relatives of patients in inpatient long-term care] (Johannes Strotbek et al., 2019). Participants were asked to indicate their level of agreement on a 6-point scale (fully agree = 0 up to fully disagree = 5). Higher values indicate lower levels of satisfaction regarding communication and contact with the clinical staff.

### Statistical Analyses

Statistical analyses were performed using IBM SPSS (version 29.0.2.0) and R software (version 4.3.0). Power analysis (G*Power, (Faul et al., 2007)) considering a two tailed t-test, an alpha level of .05, effect size (Cohens D) of 0.5, test power of 0.80 revealed a sample size of 124 individuals. The questionnaire scores assessing psychological health (PHQ), caregiver burden (ZBI), resilience (CD-RISC-10), and the quality of the communication with the patient and the clinic were compared between the two groups of relatives using between sample t-tests. The significance level was set at *p* < .05, two-tailed. For analysis of the three PHQ subscales, correction for multiple testing was applied (Bonferroni–Holm), and corrected p-values are reported. Missing items were replaced by the item means, separately for both groups. In case whole questionnaires or subscales were missing, those were excluded from the analysis, thus degrees of freedom may vary across statistical tests. Descriptive statistics show mean ± SD. Error bars in the figures show SEM. Additional analyses controlling for the type of relationship with the patient (dichotomous, partner or else), the sex of participants (dichotomous) and number of hours spent per week with care for the patient (continuous) were performed using separate ANCOVAs per dependent measure (see Supplemental Information).

## Results

### Demographics

The final sample comprised 67 RPwD (47 females; mean age: 59.55 ± SD 10.60 year) and 60 RPP (26 females; mean age: 60.82 ± SD 12.65 years). Most of the relatives were either children or partners of the patients. A smaller number of RPwD were living with the patient compared to RPP. Still, RPwD reported spending more time with informal care for the patient than RPP, as well as a higher level of care needed by the patients. Further demographic data are presented in Table 1.

### Psychological Well-Being

**Figure 2.**
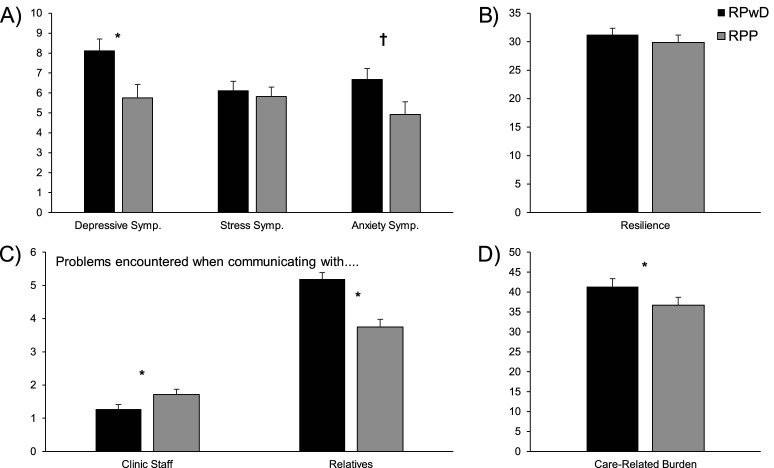
Depicted are Mean Scores (+SEM) of the Different Questionnaires and Scales. *Note:* RPwD = Relatives Patients With Dementia; RPP = Relatives Patients Psychiatry; (A) Psychological Health Assessed With the PHQ = Patient Health Questionnaire, Sub-Scales Depression, Stress and Anxiety; (B) Resilience Assessed With the CD-RISC-10 = Connor–Davidson Resilience Scale; (C) Problems Encountered when Communicating With the Clinical Staff and With the Relatives; (D) Care-related Burden Assessed With the ZBI = Zarit Burden Interview. *p < .05; †*p* = .08

RPwD reported significantly higher depression and care-related burden scores (PHQ-D: *t* (124) = 2.66, *p* = .027, *Cohen’s d* = 0.47; ZBI: *t* (123) = 3.04, *p* = .003, *Cohen’s d* = 0.55; Figure 2). Anxiety scores were also descriptively elevated among RPwD but not significantly different (PHQ-A: *t* (124) = 2.09, *p* = .08, *Cohen’s d* = 0.37; Figure 2). The two groups did not significantly differ in terms of stress symptoms (PHQ-S: *t* (120) = 0.422, *p* = 0.67, *Cohen’s d* = 0.08) and resilience (CD-RISC-10: *t* (122) = 0.81, *p* = .42, *Cohen’s d* = 0.15; Figure 2).

### Communication with Patients and Clinical Staff

RPwD reported more problems in communicating with the patients (*t* (124) = 4.81, *p* < .001, *Cohen’s d* = 0.86), while they were more satisfied with their communication with the clinical staff compared to the RPP (*t* (115) = −2.07, *p* = .04, *Cohen’s d* = − 0.38).

## Discussion

In the present study, we investigated psychological health, care-related burden, resilience, and communicative challenges among relatives of patients diagnosed with dementia (RPwD) compared to relatives of patients with a psychiatric diagnosis (RPP). Previous research already showed high psychosocial burden of relatives taking care of persons living with dementia (Cheng, 2017; Collins & Kishita, 2020; Etters et al., 2008). However, research on the relatives’ psychological health during the critical phase when patients are hospitalized in an acute geronto-psychiatric setting is lacking.

We found that RPwD reported higher levels of depression and higher care-related burden compared to the RPP. A similar pattern was found for the PHQ subscale anxiety, however, after controlling for multiple testing, the group comparison was not significant. Our results are in line with previous research reporting high psychological burden of RPwD (Cham et al., 2022; Connors et al., 2020). In line with the present findings, Epstein**-**Lubow et al. reported that taking care of a relative diagnosed with dementia, who has been recently hospitalized, represents a risk factor for the development of a mayor depression (Epstein-Lubow et al., 2012), highlighting that psychological burden persists even when the patient is not at home anymore. Further, our results corroborate previous findings, for instance by Burgstaller and colleagues, who reported high levels of worries and negative affect being very common in individuals taking care of patients with dementia (Burgstaller et al., 2018). Similarly, care-related burden (ZBI) was significantly higher in RPwD than in RPP. Scores of RPwD were on average about 41 points, which, according to the authors of the questionnaire, indicate “moderate to severe burden” (Seng et al., 2010), underscoring their particularly challenging situation. These results are in line with findings by Papastavrou et al. reporting high psychosocial burden in informal caregivers of patients with dementia, irrespective whether patients were treated at home or in a healthcare institution (Papastavrou et al., 2007). While depression was higher among the RPwD, it is important to note that psychological symptoms were also elevated in RPP, in line with previous findings (Cham et al., 2022). In our sample, the mean score of PHQ-9 assessing depression was 7.8 ± 5.0 among RPwD and 6.1 ± 5.1 among RPP, with scores ≥5 indicating mild and scores ≥10 indicating moderate depressive symptoms, respectively (Kroenke et al., 2001). Stress scores and resilience scores did not significantly differ across groups. Thus, despite a similar exposure to a wide set of psychosocial stressors and similar coping resources and strategies, it appears that mental well-being of RPwD compared to RPP is more strongly affected, likely due to the challenges inherent to the care of a patient living with dementia.

Further, both groups reported difficulties in communicating with their relatives, which, however, were significantly more pronounced in the RPwD group. Communication problems are frequent symptoms of dementia and more advanced stages of dementia are accompanied by even increasing limitations regarding verbal exchange, resulting in stress and burden for the caregivers (Banovic et al., 2018). Accordingly, psycho-educational interventions for relatives, focusing on teaching effective communication with their family member living with dementia, such as the “Communication training programs for informal caregivers of people living with dementia” described in Perkins et al. (Perkins et al., 2022) bear great potential to ameliorate the situation of patients and relatives alike.

Interestingly, both groups reported high levels of satisfaction regarding the communication with the clinical staff. Here, RPwD appeared to be even more satisfied, which might be indicative of the already high sensitivity of the health care professionals in dealing with demands and needs of patients with dementia and their relatives. As such, ward specific characteristics might be the reason for the found differences: in dementia-wards relatives are more involved in many treatment decisions, given the cognitive limitations of the patient and, additionally, clinical staff might be already sensitive and prepared to mediate difficult and complex processes of shared decision making.

### Limitations

One limitation of our study is that any information about the patients is based on the self-reports provided by the relatives, since we were not able to access the patients’ medical files. This means that we do not have any information about comorbidities (e.g., between dementia and psychiatric disorders), which might have been present in our sample. We also did not collect any information about the duration of the patients’ illness. Given that these variables could be associated with the relatives’ burden, future studies should collect this additional information. Regarding further characteristics of our samples, one must admit that a methodological strength of the present data, that is our study groups were very similar in terms of the relatives’ age, and the fact that patients were recently hospitalized in an acute psychiatric setting, at the same time makes it difficult to draw conclusions outside highly affected populations. Given the lack of a reference group consisting of age-matched individuals with older healthy relatives, the current data needs to be interpreted cautiously. It is very likely that both groups of relatives investigated here, describe more psychological strain than what is common in the general population. Therefore, future studies, incorporating carefully selected control samples, are warranted to estimate the amount of burden of RPP and RPwD. Additionally, it is important to note the relatively low response rate (only about 50%) of the relatives that initially agreed to participate in the study. This might implicate a response bias, as it is possible that there is a systematical difference between non-respondents and respondents. In addition to the high emotional burden, reasons for non-response might be the lack of time, mental and physical exhaustion, or high stress and complex organizational challenges during the hospitalization period. This may have affected the completion rate, and the underrepresentation of their perspectives may limit the generalizability of the findings. Despite the low response rate, we were able to recruit a sample size large enough for our t-test based group comparisons. Still, studies with larger samples are warranted to allow more comprehensive and stratified analyses of the many factors that might play a role in the well-being of RPwD and RPP.

### Conclusion

The present study demonstrates how taking care of an older relative with dementia, but also with a psychiatric diagnosis, impacts psychosocial well-being. Our research does not only underline the need for interventions for RPwD but also emphasizes an urgent necessity for support directed to relatives of older patients in geronto-psychiatric hospitals in general. The “family support and psychoeducation program based on the Calgary Family Intervention Model” by Sari et al. for instance is one of many options to support caregivers of (chronically) mentally ill patients, addressing psychoeducation and coping skills (Sari & Duman, 2022). In general, more research on the time course of the psychosocial status and burden of informal caregivers is necessary, to identify critical phases and the optimal time point to provide efficient demand-oriented support strategies.

## Supplemental Material

Supplemental Material - Psychological Burden and Communication Challenges Among Relatives of Older Patients With Dementia—A Cross-Sectional Study in an Acute Psychiatric Hospital Supplemental Material for Psychological Burden and Communication Challenges Among Relatives of Older Patients With Dementia—A Cross-Sectional Study in an Acute Psychiatric Hospital by Giulia Zerbini, Emanuel Wiese, Laura Muehlich, Alkomiet Hasan, Miriam Kunz, Jan Haeckert, and Philipp Reicherts in Journal of Applied Gerontology

## Data Availability

All data-analysis can be requested from the corresponding authors via giulia.zerbini@med.uni-augsburg.de
