# Supplementary material for: Psychological Burden and Communication Challenges Among Relatives of Older Patients With Dementia—A Cross-Sectional Study in an Acute Psychiatric Hospital
Source: J Appl Gerontol. 2025 Sep 8;45(8):1455–63. doi: 10.1177/07334648251372691 (PMC13323893; doi:10.1177/07334648251372691)
Supplement: Supplemental Material - Psychological Burden and Communication Challenges Among Relatives of Older Patients With Dementia—A Cross-Sectional Study in an Acute Psychiatric Hospital [file sj-pdf-1-jag-10.1177_07334648251372691.pdf]

Supplementary Information for

"Psychological burden and communication challenges among relatives of older patients with dementia – a cross-sectional study in an acute psychiatric hospital"

Additional analysis controlling for individual characteristics of the RPwD

ANCOVAs controlling for the type of relationship with the patient (dichotomous, partner or else), the sex of participants (dichotomous) and number of hours spent per week with care for the patient (continuous) revealed that RPwD reported significantly higher depressive scores (PHQ-D; $F_{1,85}$ = 4.40, p = .04) and care related burden scores (Zarit; $F_{1,86}$ = 4.39, p = .04), while we found no significant group differences for anxiety symptoms (PHQ-A; $F_{1,85}$ = 3.77, p = .06), stress symptoms (PHQ-S; $F_{1,82}$ = 0.17, p = .69) or resilience (CD-RISC-10); $F_{1,80}$ = 1.45, p = .23). Furthermore, applying the same ANCOVA logic for the analysis of communication problems experienced with the patients we found a significant difference between groups ($F_{1,86}$ = 16.92, p < .001), indicating more problems in RPwD. Regarding communication quality with clinical staff, after controlling for relationship, sex and hours spent with care work, we found no significant group difference ($F_{1,68}$ = 2.82, p = .10).
